# Complete Mitochondrial Genome Sequence of Sunflower (*Helianthus annuus* L.)

**DOI:** 10.1128/genomeA.00981-16

**Published:** 2016-09-15

**Authors:** Christopher J. Grassa, Daniel P. Ebert, Nolan C. Kane, Loren H. Rieseberg

**Affiliations:** a Department of Botany and Biodiversity Research Centre, University of British Columbia, Vancouver, BC, Canada; b Department of Biology, Indiana University, Bloomington, Indiana, USA

## Abstract

This is the first complete mitochondrial genome sequence for sunflower and the first complete mitochondrial genome for any member of *Asteraceae*, the largest plant family, which includes over 23,000 named species. The master circle is 300,945-bp long and includes 27 protein-coding sequences, 18 tRNAs, and the 26S, 5S, and 18S rRNAs.

## GENOME ANNOUNCEMENT

We present the sunflower’s (*Helianthus annuus* L.) complete mitogenome based on the male-fertile oil-seed line HA412. The annual sunflowers, including the wild *H. annuus*, are endemic to North America and are adapted to a wide variety of habitats (1). Together, they are an important model system for studying evolution and ecology, particularly reticulate evolution and the genetic and ecological processes leading to speciation (2). Sunflower is also a globally important hybrid oilseed crop (3, 4) with production valued at $20 billion annually. Mitochondrial-based cytoplasmic male sterility is employed for hybrid production.

Leaf tissue from 10-day-old seedlings was enriched for mitochondria by centrifugation, to a purity of over 99% mitochondrial DNA. DNA was sequenced on 1/48th of an Illumina lane, producing 2,727,097,000 bp of sequence data. Reads were trimmed for quality and plastid contamination (5) with Trimmomatic (6) and BWA (7), and then assembled with SOAPdenovo (8). The *de novo* assembly was digested *in silico* and aligned (9) to a previously published restriction map (10). The genome was finished by hand using Illumina and Roche 454 reads and annotated using the Mitofy software (11).

The genome’s master replication circle is 300,945 bp in length with a G+C content of 45%. It includes a large repeat 12,933 bp in length and two single-copy regions, measuring 51,681 bp and 223,398 bp. Alignments of short reads to the reference support the hypothesis that this structural configuration is rare. Rather, the genome’s predominant configuration is two equimolar circular chromosomes, each containing one copy of the large repeat and either the large or small single copy sequence (10). The genome contains a total of seven sequences at least 200 bp in length, repeated with at least 98% identity, and several other smaller repetitive sequences. A 265-bp repeat is present in three copies.

The genome includes 18 tRNA loci. Six are similar to those commonly found in plant plastids, but are not perfectly identical to those of the sunflower’s plastid. There are two *tRNA-fM* loci and one plastid-like *tRNA-M* locus. The *tRNA-I* locus contains a CAU anticodon, suggesting that it is modified posttranscription. The 26S, 5S, and 18S rRNAs are present. The genome includes at least 27 protein-coding sequences. Two genes, *rps3* and *mttB*, begin with an ATT start codon. Five sequences are homologous to the sunflower’s plastid genome.

Just 25,611 bp, approximately 8.5%, of the genome could be functionally annotated. An additional 8,149 bp appear to be pseudogenes in various states of decay. The sunflower’s mitogenome is repetitive and sparsely populated with genes. This is typical for a plant, but stands in stark contrast with the streamlined mitogenomes of animals. This reference is expected to facilitate the guided assembly of the mitogenomes of hundreds of sequenced sunflower accessions, as well as other *Asteraceae*, and will be an important resource for plant breeders and evolutionary biologists.

### Accession number(s).

This organelle genome project has been deposited in GenBank under the accession number KF815390. The version described in this paper is the first version, KF815390.1.
